# *Caralluma fimbriata* Extract Improves Vascular Dysfunction in Obese Mice Fed a High-Fat Diet

**DOI:** 10.3390/nu16244296

**Published:** 2024-12-12

**Authors:** Venkata Bala Sai Chaitanya Thunuguntla, Laura Kate Gadanec, Catherine McGrath, Joanne Louise Griggs, Puspha Sinnayah, Vasso Apostolopoulos, Anthony Zulli, Michael L. Mathai

**Affiliations:** 1 Institute for Health and Sport, Victoria University, Melbourne, VIC 3030, Australia; venkatabalasaichaitanya.thunuguntla@vu.edu.au (V.B.S.C.T.); laura.gadanec@live.vu.edu.au (L.K.G.); catherine.mcgrath1@live.vu.edu.au (C.M.); joanne.griggs@vu.edu.au (J.L.G.); puspha.sinnayah@vu.edu.au (P.S.); vasso.apostolopoulos@rmit.edu.au (V.A.); 2 School of Health and Biomedical Sciences, RMIT University, Melbourne, VIC 3083, Australia

**Keywords:** *Caralluma fimbriata* extract, high-fat diet, lorcaserin, obesity, vascular dysfunction, natural product, bio-active compounds

## Abstract

Background: Obesity is a risk factor for developing cardiovascular diseases (CVDs) by impairing normal vascular function. Natural products are gaining momentum in the clinical setting due to their high efficacy and low toxicity. *Caralluma fimbriata* extract (CFE) has been shown to control appetite and promote weight loss; however, its effect on vascular function remains poorly understood. This study aimed to determine the effect that CFE had on weight loss and vascular function in mice fed a high-fat diet (HFD) to induce obesity, comparing this effect to that of lorcaserin (LOR) (an anti-obesity pharmaceutical) treatment. Methods: C57BL/6J male mice (*n* = 80) were fed a 16-week HFD to induce obesity prior to being treated with CFE and LOR as standalone treatments or in conjunction. Body composition data, such as weight gain and fat mass content were measured, isometric tension analyses were performed on isolated abdominal aortic rings to determine relaxation responses to acetylcholine, and immunohistochemistry studies were utilized to determine the expression profiles on endothelial nitric oxide synthase (eNOS) and cell stress markers (nitrotyrosine (NT) and 78 kDa glucose-regulated protein (GRP78)) in the endothelial, medial and adventitial layers of aortic rings. Results: The results demonstrated that CFE and CFE + LOR treatments significantly reduced weight gain (17%; 24%) and fat mass deposition (14%; 16%). A HFD markedly reduced acetylcholine-mediated relaxation (*p* < 0.05, *p* < 0.0001) and eNOS expression (*p* < 0.0001, *p* < 0.01) and significantly increased NT *(p* < 0.05, *p* < 0.0001) and GRP78 (*p* < 0.05, *p* < 0.01, *p* < 0.001). Obese mice treated with CFE exhibited significantly improved ACh-induced relaxation responses, increased eNOS (*p* < 0.05, *p* < 0.01) and reduced NT (*p* < 0.01) and GRP78 (*p* < 0.05, *p* < 0.01) expression. Conclusions: Thus, CFE alone or in combination with LOR could serve as an alternative strategy for preventing obesity-related cardiovascular diseases.

## 1. Introduction

Cardiovascular diseases (CVDs), clinically defined as any pathology effecting the heart and/or vasculature (e.g., atherosclerosis, coronary artery disease (CAD), cerebrovascular disease and peripheral artery disease) [1] are the leading cause of global mortality and morbidity, being responsible for 19.1 million deaths in 2022 [2]. Hypertension [3], obesity [4], cigarette smoking [5], hyperhomocysteinemia [6], a sedentary lifestyle [7], inflammation [8], type-2 diabetes and insulin resistance [9] are some of the risk factors associated with CVDs.

Obesity is a complex disorder of energy imbalance and represents a widespread epidemic, as it is estimated that 50% of men and 55% of women, globally, are categorized as overweight or obese [10]. In 2022, nearly 890 million people worldwide were classified as obese, reflecting more than a twofold increase in global obesity rates since 1990. This alarming trend highlights the growing public health challenge posed by obesity, which is a major risk factor for numerous non-communicable diseases, particularly cardiovascular diseases (CVD), type 2 diabetes, certain cancers, and respiratory disorders (https://www.who.int/news-room/fact-sheets/detail/obesity-and-overweight, accessed on 27 November 2024). Excessive caloric intake accompanied by a sedentary lifestyle results in augmented subcutaneous and visceral adipose tissue storage [4]. Obesity occurs when the percentage of body fat is increased to an extent that it impairs health and well-being, and the World Health Organization and American Heart Association use the body mass index (BMI; body weight kg/height m^2^) to determine if an individual is considered underweight (BMI: <18.5 kg/m^2^); normal (18.5–24.9 kg/m^2^); overweight (25.0–29.9 kg/m^2^); category I obese (30.0–34.9 kg/m^2^); category II obese (35.0–39.9 kg/m^2^); or category III obese (>40.0 kg/m^2^) [4,10,11]. Shockingly, a one-point increase in a person’s BMI above the normal weight range is linked to a 10% higher risk of developing atherosclerosis and CAD [4].

Importantly, obesity has been linked with endothelial dysfunction, which is the earliest vascular abnormality related to atherosclerosis and other CVDs [12], as well as a complex network of immunological and inflammatory responses [13]. Studies involving isolated subcutaneous fat arterioles and brachial arteries from obese individuals have reported diminished nitric oxide (NO) production, reduced endothelium nitric oxide synthase (eNOS) protein expression and activity, and impaired acetylcholine (ACh)- and endothelium-dependent relaxation [14,15,16]. Alarmingly, a modest gain of visceral fat among healthy young adults leads to impaired endothelial function, even without any noticeable changes in blood pressure [17]. Furthermore, obesity and hyperlipidemia have also been linked to upregulated expression of molecules involved in cellular stress pathways, including endoplasmic reticulum stress, unfolded protein response and mitophagy [18], oxidative stress and redox imbalance [19], as well as nitrosative stress [20].

For over a century, the development of anti-obesity pharmaceuticals has concentrated on facilitating weight loss by targeting feeding behavior and appetite, enhancing insulin resistance, and restoring glucose homeostasis [21]. However, translation into human trials has been challenging due to unwanted drug–drug interactions, harmful off target effects and adverse events, which are a major public health concern [22]. For example, lorcaserin (LOR) was initially marketed for weight loss and management in the clinical setting by decreasing appetite by interfering with the serotonin receptor-2c receptor (5HT2cR) [23]; however, it was withdrawn in February, 2020, following a Drug Safety Communication issued by the Food and Drug Administration, which reported a possible increased risk of pulmonary, colon and pancreatic cancers [24]. Natural products for the treatment of obesity have continued to gain popularity as an alternative to synthetic drugs due to their low toxicity and high efficacy [24]. *Caralluma fimbriata* extract (CFE), a natural product derived from an edible succulent native to India that belongs to the *Asclepiadaceae* family, has shown promise in clinical trials by promoting waist circumference reduction and weight loss via suppressing appetite [25,26]. Moreover, CFE may have anti-atherosclerotic abilities [27], resulting in reduced CVD risk [28]. Our previous studies demonstrated that the hydroethanolic extract of CFE treatment in SNORD116 mice resulted in a significant upregulation of alpha-melanocyte-stimulating hormone (α-MSH) signaling, while treatment with the selective 5HT2cR antagonist, SB 242084, led to a reduction in α-MSH expression [29]. These findings further corroborate that 5HT2cR activation initiates downstream signaling pathways involved in satiety, mediated through α-MSH [30].

Herein, we determined the effects of CFE and low dose LOR on weight loss, fat deposition and vascular function in obese mice fed a high-fat diet (HFD). The EchoMRI-900 was used to measure fat and lean mass distribution, while isometric tension myography organ bath studies were conducted on the abdominal aorta to evaluate ACh-dependent relaxation responses. Additionally, semi-quantitative immunohistochemistry analysis was performed to investigate the expression of markers related to vascular function (such as eNOS), endoplasmic reticulum stress (specifically, the 78 kDa glucose-regulated protein, GRP78), and nitrosative stress (i.e., nitrotyrosine, NT) in the endothelium, media, and adventitia of the abdominal aorta.

## 2. Materials and Methods

### 2.1. Materials

Rabbit monoclonal anti-CD31 (Cat#ab182981) antibody was purchased from Abcam (Melbourne, VIC, Australia); 3,3′-diaminobenzidine (DAB) substrate kit (Cat#550880) was purchased from Becton Dickinson Biosciences (Franklin Lakes, NJ, USA); rabbit monoclonal NT (D2W9T) (Cat#92212) antibody was purchased from Cell Signaling Technology (Danvers, MA, USA); rabbit polyclonal GRP78 antibody (Cat#GTX127934) was purchased from GeneTex (Irvine, CA, USA); lorcaserin and the hydro-ethanolic extract of *Caralluma fimbriata* were provided as gifts by Gencor Pacific (Austin, TX, USA); gelatine was purchased from McKenzie’s Foods (Melbourne, VIC, Australia); and ACh chloride (Cat#A6625), saccharine (Cat#109185), and U4669 (Cat#538944) were purchased from Sigma Aldrich (St. Louis, MO, USA).

*Caralluma fimbriata* extract (CFE): The aerial parts of the plant, including stems, leaves and flowers, are processed by drying and pulverization before extraction using a hydroethanolic solvent (70% water, 30% ethanol). After extraction, the solvent is evaporated, and the residue is sifted into a fine powder. The extract primarily consists of pregnane glycosides, which represent approximately 25% (*w*/*w*) of the final product [31].

### 2.2. Animals and Ethical Approval

Male C57BL/6J (*n* = 80) at 5 weeks of age were purchased from the Animal Resource Centre (Perth, WA, Australia) and were housed individually at the Victoria University Werribee Campus Animal Facility. To determine the appropriate sample size required for body weight and fat content, a power analysis was conducted using G*Power (version 3.1.9.4) [32]. An F-test was selected for an ANOVA with repeated measures (power analysis: post hoc), within factors (control vs. treatment groups). The following parameters were used for the power analysis: a large effect size (f = 0.40), an alpha level of 0.05, and a target power of 0.80. The required sample size was determined to be 16 animals per group, with 5 groups (control + 4 treatments), resulting in a total of 80 animals. This sample size would provide 80 % power to detect a large effect size at the 0.05 significance level. Upon arrival, animals were given a 7-day acclimatization period and were maintained at a constant temperature of 22 °C and relative humidity levels between 40 and 70%. The animals were kept on a 12 h day/night circadian rhythm cycle, and food and water were supplied ad libitum. All experimental procedures were conducted in accordance with the National Health and Medical Research Council’s Australian Code of Practice for the Care and Use of Animals for Scientific Purposes’ (8th edition, 2013; https://www.nhmrc.gov.au/about-us/publications/australian-code-care-and-use-animals-scientific-purposes, accessed on 12 May 2019). Animal experiments were approved by the Victoria University Animal Ethics Committee under approval number VUAEC #19/007. Following acclimatization, the individual weights of all mice were recorded to enable randomization into each treatment group. By the conclusion of the acclimatization period, the treatment groups exhibited similar body weights.

### 2.3. Diet Protocol and Jelly Treatment

At 6 weeks of age, 16 mice per group were randomly assigned to the following conditions: control, HFD, CFE, LOR and CFE + LOR (total *n* = 80). The randomization was based on individual weights, measured at the end of the acclimatization by using excel “Rand()” command. To eliminate confounders, the animals were housed in the same room and all treatments were performed at the same time. Control (Cat#SF13-081) and high-fat (Cat#SF04-001) feeds were purchased from Specialty Feeds (Glen Forrest, WA, Australia) [33]. During the first 8 weeks of the study, animals were monitored weekly for food intake and weight gain. Mice were then housed individually, and CFE and LOR treatments were administered as jelly cubes (each cube weighed 1% of the total body weight of the animal) during HFD induction. The desired concentrations of CFE and LOR were infused into the jelly cubes (Table 1), which were administered daily, 2 h prior to the dark cycle. The selective deletion of Snord116 in the mouse results in a phenotype that includes persistent low birth weight, increased energy expenditure, increased weight gain during early adulthood, and hyperphagia. These findings underscore the critical role of Snord116 in the regulation of feeding behavior and energy homeostasis [34]. Our team’s prior investigations using the Snord116 mouse model examined the appetite suppression impact of CFE at a dose of 100 mg/kg body weight per day [29]. A LOR concentration of 5 mg/kg body weight was determined as the minimal dose necessary to create appetite management. The required dosage quantities were infused into the gelatine mix, then saccharine (Hemesetas, 0 Kcal of energy, 0.01% salt) was added [29]. All mice underwent a 5-day pre-training period with jelly rewards before the treatments [35]. The food intake of each individual mouse was measured weekly by subtracting the leftover food from the designated amount provided [36].

### 2.4. Body Weight and EchoMRI

All mice were weighed weekly, and the fat and lean mass contents were measured using an EchoMRI-900 (Echo-MRITM 900, Houston, TX, USA) [37] both pre- and post-treatment, according to the manufacturer’s protocol [38]. The percentages of fat and lean mass were computed in relation to body weight.

### 2.5. Humane Dispatch and Isometric Tension Analysis

The animals were first anesthetized using isoflurane (2.5%) and were humanely euthanized via cardiac puncture. The abdominal aortae were then isolated, cleaned of adipose and connective tissue, and dissected into 3 mm rings. The rings were immediately and sequentially placed into adjacent organ baths (OB8 Zultek Engineering, VIC, Australia) filled with Krebs-Henseleit solution (118 mM, NaCl; 4.7 mM KCl; 1.2 mM MgSO_4_·7H_2_O; 1.2 mM KH_2_PO_4_; 25 mM NaHCO_3_; 11.7 mM glucose; and 1.25 mM CaCl_2_) (pH 7.4). To mimic a physiologically relevant environment, the organ baths were maintained at 37 °C and continuously bubbled with carbogen (95% oxygen: 5% carbon dioxide) [39]. The rings were acclimatized for 30 min and then mounted between two metal organ hooks, attached to a force displacement transducer and stretched to 0.2–0.4 g [39]. The rings were rested for a further 30 min and were re-stretched [39]. To determine the relaxation ability of the abdominal aortae, the rings were sub-maximally pre-contracted with the thromboxane analogue, U44619 [2 × 10^–5^ M], to observe at least a doubling in tension [39] (Supplementary Figure S1). When a plateau was reached, the rings were relaxed using an ACh dose–response [10^–8^ M–10^–5^ M] [40] (Supplementary Figure S1).

### 2.6. Immunohistochemistry Technique and Semi-Quantitative Analysis

Following isometric tension myography studies, the abdominal aortae were immediately placed into 4% paraformaldehyde (pH 7.4) for 24 h, transferred to embedding cassettes and refrigerated (4 °C) in 1× phosphate-buffered saline solution (pH 7.4) for 24 h. The tissues were then processed concomitantly in paraffin in paraffin using a Thermo Scientific Spin Tissue Processor Microm STP 120 (Scoresby, VIC, Australia). After tissue processing, the vessels were vertically embedded into blocks of paraplast and cut into 5 µm sections using a manual microtome. The ribbon sections were then placed into a 45 °C water bath containing dissolved gelatin, allowed to expand to original size, mounted onto slides and dried in an oven at 37 °C for 3 days. The tissues were de-paraffined and rehydrated using a xylene and ethanol gradient, followed by Tris buffer (pH 7.4), as previously established in this laboratory [40]. Non-specific antigen sites were blocked using 1% goat serum for 60 min. The antibodies were added to the tissue (1:100) and incubated for 24 h. The tissues were then incubated with secondary antibodies for 60 min, and DAB was added to the tissues for 15 min to stain for the antigen of interest. The tissues were then counterstained with hematoxylin, dehydrated, mounted and prepared for the quantification analysis.

To determine the expression of proteins in the endothelium, media and adventitia, 3–5 photographs per ring were taken at ×400 and ×1000 magnification under Olympus BX50 microscope (Olympus Life Science, Notting Hill, VIC, Australia). Each image was loaded into the Micro Computer Imaging Device (Interfocus, Linton, Cambridge, UK) analysis program, and the ribbon tool was selected to trace the endothelium and the outline tool was used to trace the media and adventitia [40]. A specific range of color intensity and hue was selected to detect the expression of the DAB stain [40]. The color intensity and proportional area were recorded and averaged for images of each individual ring [40]. The values were recorded in a spreadsheet and the proportional intensity was calculated using the following equation: proportional intensity = (1/color intensity) × proportional area [40]. The proportional intensity was then averaged for each tissue [40]. Due to loss of tissue during paraffin processing, block cutting and slide mounting, an *n* = 3–4 for immunohistochemistry studies was retrievable.

### 2.7. Statistical Analysis

GraphPad prism version 9.5.1 was used to analyze data for the experiments conducted in this study. A two-way ANOVA and one-way ANOVA followed by Sidak’s multiple comparisons post hoc test was performed to determine significance from isometric tension analysis studies and immunohistochemistry, respectively. The significant *p* value was set at *p* < 0.05, and all data were represented as mean ± standard error of the mean (SEM). No animals or data points were excluded from the study during analysis.

## 3. Results

### 3.1. CFE Reduces Weight Gain in Mice Fed a HFD

**Figure 1:**
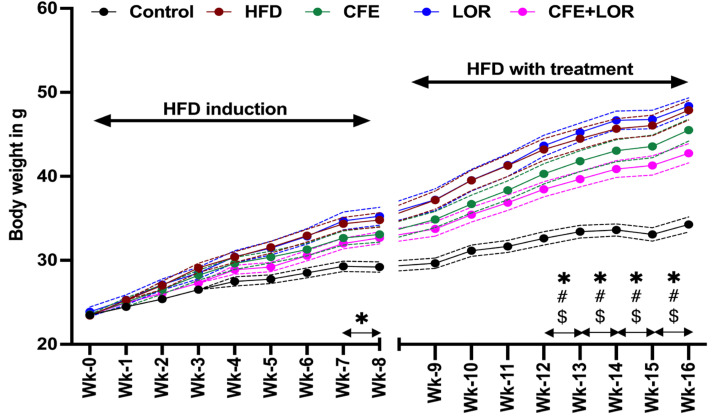
Body weights during the 16-week study. Mice body weights are shown each week during HFD induction from week 0 to week 8. The body weights from week 9 to week 16 represent the treatment period with CFE and/or LOR. All the data are derived from *n* = 16/group and values plotted are presented as the mean ± SEM. The statistical significance * *p* ≤ 0.05 represents the comparison between the control and the HFD groups; ‘#’ represents HFD with CFE + LOR, and ‘$’ represents LOR with CFE + LOR. Abbreviations: CFE, *Caralluma fimbriata* extract; HFD, high-fat diet; LOR, lorcaserin; SEM, standard error of the mean.

**Figure 2:**
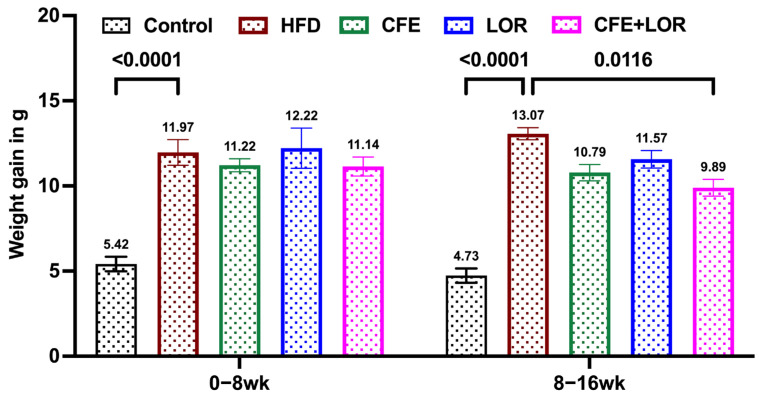
Change in weight gain during the 16-week study. The weight gain of all the groups during the HFD induction and treatment periods are represented individually. Each group had an *n* = 16, and the values plotted are the mean ± SEM. Significance is set at *p* ≤ 0.05. Abbreviations: CFE, *Caralluma fimbriata* extract; HFD, high-fat diet; LOR, lorcaserin; SEM, standard error of the mean.

The mice fed a HFD had a marked increase in body weight during the initial 8 weeks when compared to the mice fed a control diet (HFD: *p* = 0.004; CFE: *p* = 0.01; LOR: *p* = 0.01 and CFE + LOR: *p* = 0.01) (Figure 1). The combination treatment with CFE + LOR resulted in a significant decline in body weight gain from weeks 12 (*p* = 0.03) to 16 (*p* = 0.02), when compared to the HFD-fed mice. Similarly, a significant reduction in body weight gain from week 12 (*p* = 0.01) to 16 (*p* = 0.014) was observed when comparing CFE + LOR-treated mice to the LOR-treated mice (Figure 1). The HFD-fed mice in all the groups gained significant weight when compared to the mice fed a control diet during 0–8 weeks of diet induction (*p* < 0.0001) (Figure 2). Additionally, the most significant reduction in weight gain was in the animals treated with CFE + LOR (Figure 2) in comparison to the HFD-fed group. Interestingly, CFE (10.79 g) alone showed a greater reduction in weight gain when compared to LOR (11.57 g) (Figure 2).

### 3.2. CFE Reduces Fat Mass

**Figure 3:**
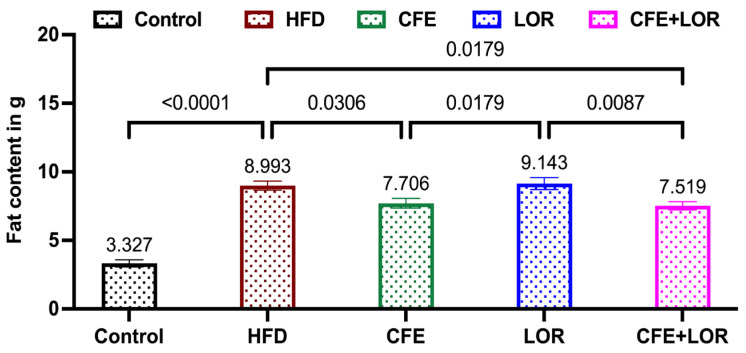
Change in fat content during the treatment period (8 weeks): EchoMRI-based fat mass measurement difference between weeks 8 and 16. Each group consisted of *n* = 16 mice, and the values plotted are represented as the mean ± SEM. Significance is set at *p* ≤ 0.05. Abbreviations: CFE, *Caralluma fimbriata* extract; HFD, high-fat diet; LOR, lorcaserin; SEM, standard error of the mean.

The EchoMRI-derived fat composition represents the difference in fat deposition between the pre- and post-treatment time points. The fat deposition in HFD mice was significantly elevated when compared to the control diet (*p* < 0.0001) (Figure 3). In contrast, CFE (7.70 g, *p* = 0.03)-treated mice and the CFE + LOR (7.51 g, *p* = 0.02)-treated mice had markedly reduced fat content when compared to the HFD-fed mice (8.99 g) (Figure 3). Surprisingly, HFD (8.99 g) and LOR (9.14 g) groups demonstrated a similar fat content. Furthermore, the LOR-treated animals had significantly more fat content when compared to the CFE (*p* = 0.02) and the CFE + LOR (*p* = 0.01) groups (Figure 3).

**Figure 4:**
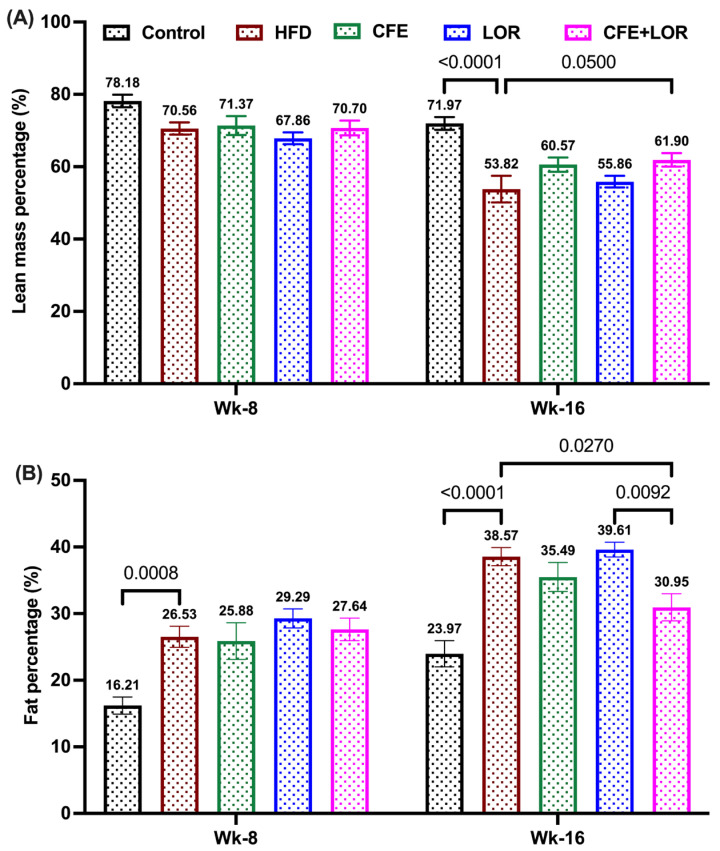
Fat and lean mass percentage with respect to bodyweight. Lean mass percentage (**A**) and fat mass percentage (**B**) during the 8th and 16th weeks with respect to bodyweights. Each group included *n* = 16 mice and the values plotted are the mean ± SEM. Significance was set at *p* ≤ 0.05. Abbreviations: CFE, *Caralluma fimbriata* extract; HFD, high-fat diet; LOR, lorcaserin; SEM, standard error of the mean; Wk, weeks.

The lean mass was significantly (*p* < 0.0001) reduced in the HFD-fed group when compared to the mice fed a control diet (Figure 4A). On the other hand, the fat mass was significantly increased in the HFD-fed mice when compared to the control group (*p* < 0.0001) (Figure 4B). The post-treatment lean mass in the HFD-fed group (53.82%) significantly declined when compared to LOR treatment (61.9%) (*p* = 0.05) (Figure 4A). Similarly, the fat mass percentage was reduced in the CFE + LOR-treated animals (30.95%; *p* = 0.03) in comparison to the HFD-fed mice (38.57%) and LOR treatment (39.61%; *p* = 0.01) (Figure 4A).

### 3.3. A HFD Reduces Relaxation to ACh and Is Imrpoved by CFE

**Figure 5:**
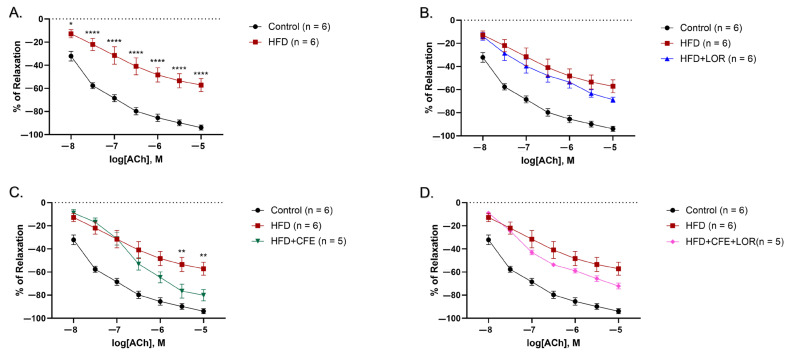
Relaxation responses of abdominal aortic rings to ACh dose–response. (**A**) The aortic rings from the mice fed a 16-week HFD had significantly reduced ability to relax to ACh when compared to the mice fed a control diet (mean ± SEM, *p* < 0.05, *p* < 0.0001). (**B**) Treatment with LOR was unable to improve relaxation to ACh in mice fed a HFD (mean ± SEM). (**C**) CFE was able to significantly enhance relaxation responses to ACh in mice fed a HFD (mean ± SEM, *p* < 0.05, *p* < 0.01, *p* < 0.001). (**D**) The combination treatment of CFE + LOR was unable to significantly increase ACh-mediated relaxation (mean ± SEM). Abbreviations: ACh, acetylcholine; CFE, *Caralluma fimbriata*; HFD, high-fat diet; LOR, lorcaserin; SEM, standard error of the mean. Key: * = *p* < 0.05, ** = *p* < 0.01 and **** = *p* < 0.0001.

A 16-week HFD significantly diminished relaxation capacity in the abdominal aortic rings to ACh at all doses: from ACh 10^–8^ M (control: −32.14 ± 4.18% vs. HFD: −11.67 ± 4.05%, *p* = 0.0165) to ACh^–5^ M (control: 98.89 ± 2.19% vs. HFD: −54.31 ± 5.86%, *p* < 0.0001), when compared to the control-fed mice (Figure 5A). Treatment with LOR was unable to improve relaxation responses of the abdominal aortae from the HFD-fed mice (Figure 5B); however, the aortic rings from the mice treated with CFE displayed markedly enhanced relaxation responses to Ach, from ACh^–6.5^ M (HDF: −36.42 ± 7.17% vs. HDF + CFE: −56.22 ± 8.50%, *p* = 0.0041) to ACh^–5^ M (HFD vs. HFD + CFE: −85.07 ± 6.36%, *p* = 0.0045) (Figure 5C). Interestingly, no significant differences were observed between the relaxation responses of the HFD-fed mice and the CFE + LOR combination treatment; however, a slight improvement was noted (Figure 5D).

### 3.4. HFD-Induced Obesity Reduces eNOS and Increases GRP78 and NT Expression in Abdominal Aorta: Improvements Achieved with CFE Treatment

**Figure 6:**
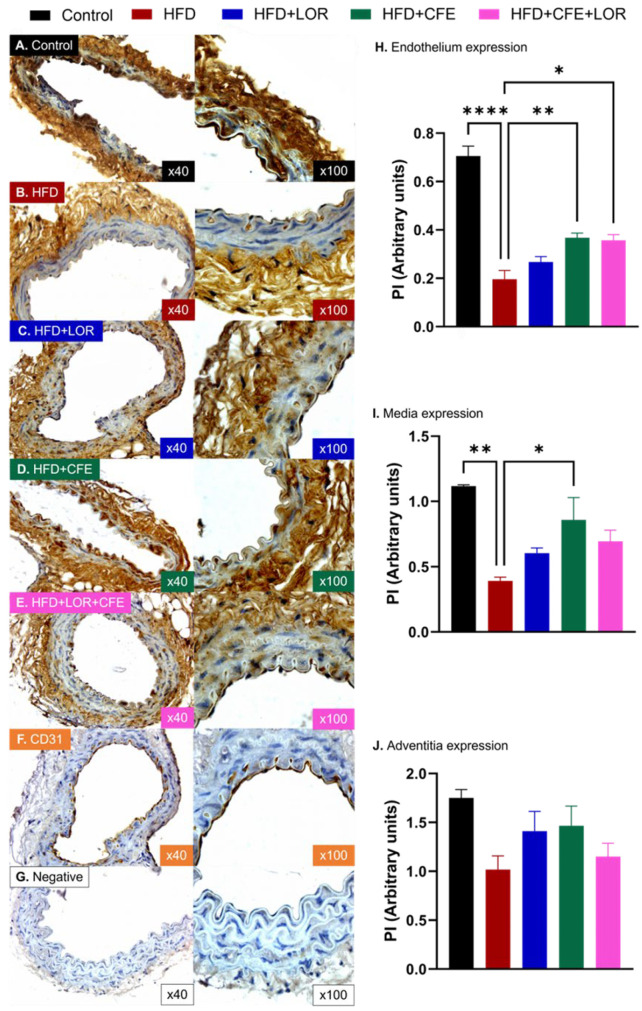
eNOS expression in different layers of mouse abdominal aorta. Immunohistochemical localization of eNOS in the endothelium, media and adventitia of abdominal aortae from (**A**) mice fed a control diet (*n* = 3); (**B**) mice fed a 16-week HFD to induce obesity (*n* = 4); (**C**) mice fed a HFD and treated with LOR (*n* = 4); (**D**) mice fed a HFD and treated with CFE (*n* = 4); and (**E**) mice fed a HFD and treated with CFE and LOR (*n* = 3). (**F**) Positive control slides were stained with the anti-CD31 antibody, while the (**G**) negative control slides were incubated without the primary antibody. (**H**) Obese mice fed a 16-week HFD had markedly reduced endothelial eNOS expression (mean ± SEM, *p* < 0.0001), which was significantly increased by the CFE treatment either alone (mean ± SEM, *p* < 0.01) or in combination with LOR (mean ± SEM, *p* < 0.05). (**I**) Media expression of eNOS in HFD-fed mice was significantly decreased (mean ± SEM, *p* < 0.01) and was enhanced by CFE treatment (mean ± SEM, *p* < 0.05). (**J**) Although not statistically significant, adventitial expression of eNOS was decreased by the HFD and was increased across all treatment groups. Abbreviations: CFE, *Caralluma fimbriata*; eNOS, endothelial nitric oxide synthase; HFD, high-fat diet; LOR, lorcaserin; PI, proportional intensity; SEM, standard error of the mean. Key: * = *p* < 0.05, ** = *p* < 0.01 and **** = *p* < 0.0001.

The mice fed a 16-week HFD to induce obesity exhibited significantly lower expression of eNOS in the endothelium (control: 0.20 ± 0.04 PI vs. HFD: 0.71 ± 0.05 PI, *p* < 0.0001) (Figure 6A,B,H) and media (control: 1.12 ± 0.01 PI vs. HFD: 0.39 ± 0.14 PI, *p* = 0.0012) (Figure 6A,B,I) of the abdominal aorta when compared to the control mice, fed a standard chow diet. A reduction in eNOS expression was also observed in the adventitia; however, no significance was reported (Figure 6A,B,J). Treatment with CFE was able to markedly increase the eNOS expression in the endothelium (HFD vs. HFD + CFE: 0.37 ± 0.02 PI, *p* = 0.0053) (Figure 6B,D,H) and media (HFD vs. HFD + CFE: 0.86 ± 0.17 PI, *p* = 0.0204) (Figure 6B,D,I) but not in the adventitia (Figure 6B,D,J). While the combination of LOR and CFE was able to enhance eNOS expression in the endothelium (HFD vs. HFD + CFE + LOR: 0.36 ± 0.02 PI, *p* = 0.0151) (Figure 6B,E,H), no other significant differences were observed in the mice treated with LOR (Figure 6B,C,H–J) alone or in combination with CFE (Figure 6B,E,I,J). 

**Figure 7:**
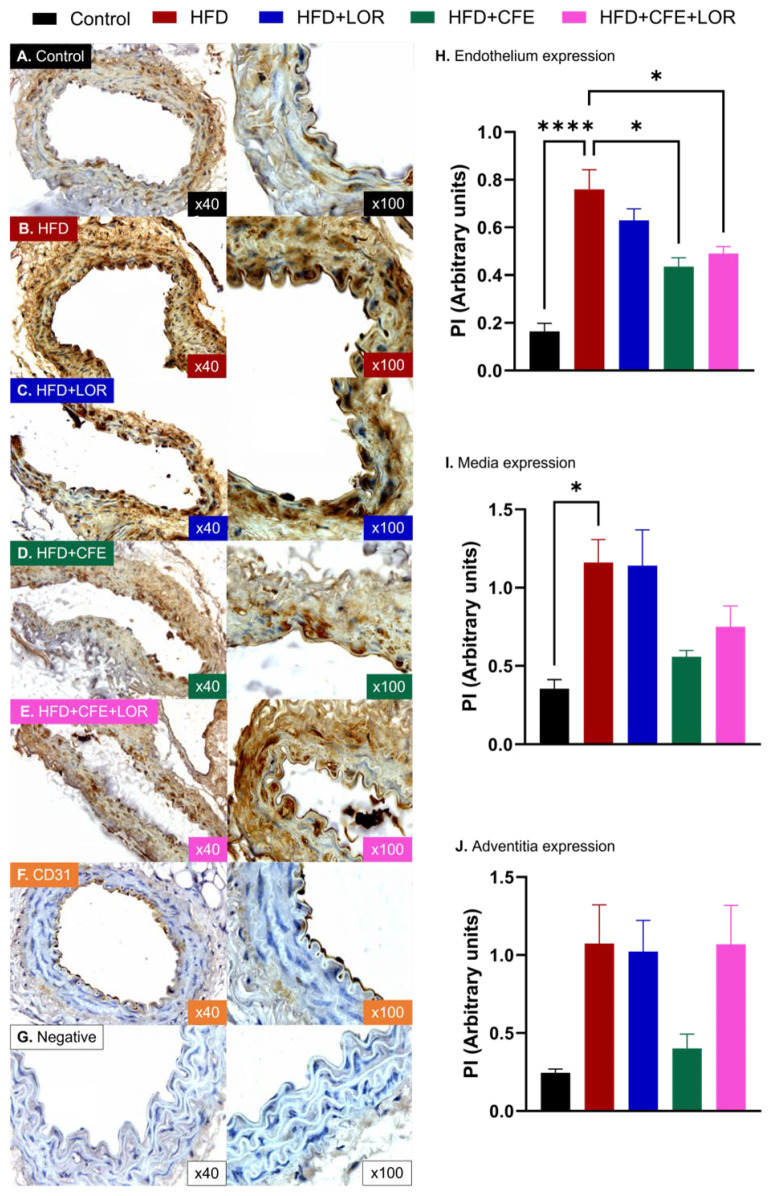
NT expression in different layers of mouse abdominal aorta. Immunohistochemical localization of NT in the endothelium, media and adventitia of abdominal aortae from (**A**) mice fed a control diet (*n* = 3); (**B**) mice fed a 16-week HFD to induce obesity (*n* = 4); (**C**) HFD-fed mice treated with LOR (*n* = 3); (**D**) HFD-fed mice treated with CFE (*n* = 3); and (**E**) HFD-fed mice treated with CFE and LOR (*n* = 3). Positive control slides were stained with ant-CD31 antibody (**F**) and negative control slides were not incubated with the primary antibody (**G**). (**H**) Obese mice fed a 16-week HFD had markedly increased endothelial NT expression (mean ± SEM, *p* < 0.0001), which was significantly reduced by CFE treatment, either alone (mean ± SEM, *p* < 0.05) or in combination with LOR (mean ± SEM, *p* < 0.05). (**I**) Media expression of NT in HFD mice was significantly elevated (mean ± SEM, *p* < 0.05). Although there were no significant differences, the CFE and LOR + CFE treatments appeared to reduce NT expression. (**J**) There were no significant differences in adventitial NT expression between groups; however, the control and the HFD-fed mice treated with CFE demonstrated lower NT levels. Abbreviations: CFE, *Caralluma fimbriata*; HFD, high-fat diet; LOR, lorcaserin; NT, nitrotyrosine; PI, proportional intensity; SEM, standard error of the mean. Key: * = *p* < 0.05 and **** = *p* < 0.0001.

NT expression was markedly upregulated in the endothelium (control: 0.16 ± 0.03 PI vs. HDF: 0.76 ± 0.08 PI, *p* < 0.0001) (Figure 7A,B,H) and media (control: 0.36 ± 0.06 PI vs. HFD: 1.16 ± 0.15 PI, *p* = 0.0111) (Figure 7A,B,I) in obese mice fed a HFD, when compared to the control mice. While an increase in NT expression was demonstrated in the adventitia, no significance was noted (Figure 7A,B,J). Mice treated with CFE displayed significantly lower NT expression in the endothelium (HFD vs. HFD + CFE: 0.44 ± 0.04 PI, *p* < 0.05) (Figure 7B,D,H). Although not statistically significant, NT expression was also reduced in the media (Figure 7B,D,I) and adventitia (Figure 7B,D,J) of obese mice. Treatment with LOR (Figure 7C,H–J) and CFE + LOR (Figure 7E,H–J) failed to significantly reduce NT expression in all arterial layers.

**Figure 8:**
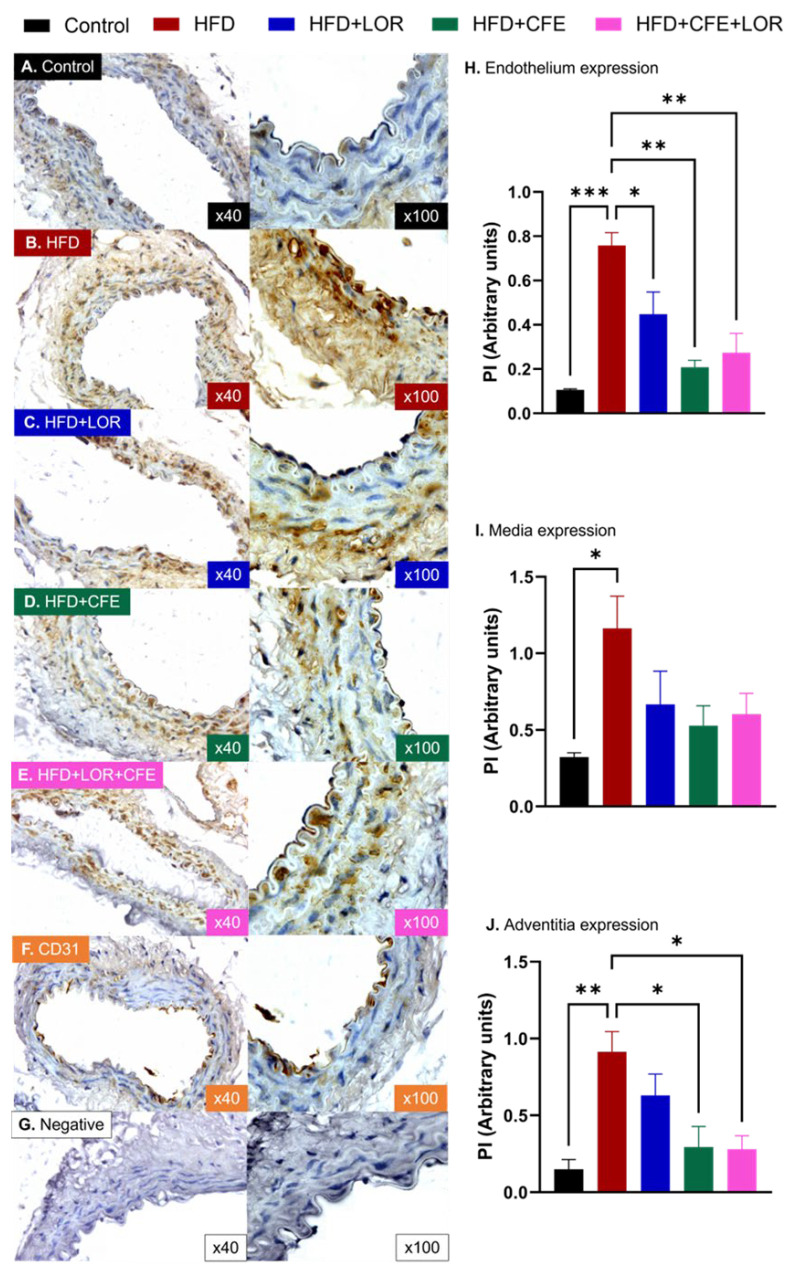
GRP78 expression in different layers of mouse abdominal aorta. Immunohistochemical localization of GRP78 in the endothelium, media and adventitia of abdominal aortae from (**A**) mice fed a control diet (*n* = 3); (**B**) mice fed a 16-week HFD to induce obesity (*n* = 3); (**C**) HFD-fed mice treated with LOR (*n* = 3); (**D**) HFD-fed mice treated with CFE (*n* = 3) and (**E**) HFD-fed mice treated with CFE and LOR (*n* = 3). The positive control slides were stained with anti-CD31 antibody (**F**) and the negative control slides were not incubated with the primary antibody (**G**). (**H**) Obese mice fed a 16-week HFD had markedly increased endothelial GRP78 expression (mean ± SEM, *p* < 0.001), which was significantly reduced by LOR (mean ± SEM, *p* < 0.05) treatment, CFE (mean ± SEM, *p* < 0.01) treatment or their combination (mean ± SEM, *p* < 0.01). (**I**) Media expression of GRP78 in HFD mice was significantly elevated (mean ± SEM, *p* < 0.05). Although there were no statistically significant differences, the CFE, LOR and LOR + CFE treatments reduced its expression. (**J**) GRP78 expression in the adventitia was significantly augmented in the HFD mice (mean ± SEM, *p* < 0.01), and both CFE (mean ± SEM, *p* < 0.05) and LOR + CFE (mean ± SEM, *p* < 0.05) were able to markedly reduce this upregulation. Abbreviations: CFE, *Caralluma fimbriata*; HFD, high-fat diet; GRP78, 78 kDa glucose-regulated protein; LOR, lorcaserin; PI, proportional intensity; SEM, standard error of the mean. Key: * = *p* < 0.05, ** = *p* < 0.01 and *** = *p* < 0.001.

Similarly, the obese mice displayed significantly higher levels of GRP78 in the endothelium (CD: 0.11 ± 4.63 × 10^–3^ PI vs. HDF: 0.76 ± 0.06 PI, *p* = 0.0003) (Figure 8A,B,H), media (CD: 0.32 ± 0.03 PI vs. HFD: 1.16 ± 0.21 PI, *p* = 0.0254) (Figure 8A,B,I) and adventitia (CD: 0.15 ± 0.06 PI vs. HFD: 0.91 ± 0.13, *p* = 0.0024) (Figure 8A,B,J). Both CFE and CFE + LOR treatments were able to reduce GRP78 in the endothelium (HFD vs. HFD + CFE: 0.21 ± 0.03 PI vs. HFD + CFE + LOR: 0.27 ± 0.09, *p* = 0.0012 and *p* = 0.0030) (Figure 8B,D,E,H) and adventitia (HFD vs. HFD + CFE: 0.29 ± 0.13 PI vs. HFD + CFE + LOR: 0.28 ± 0.09, *p* = 0.0172 and *p* = 0.0149) (Figure 8B,D,E,J), while LOR treatment was only able to reduce endothelial expression of GRP78 (HFD vs. HFD + LOR: 0.45 ± 0.06, *p* = 0.0490) (Figure 8B,C,H).

## 4. Discussion

This study is the first to demonstrate that CFE treatment provides CVD protection in obese mice fed a HFD by enhancing ACh-dependent relaxation, reducing cell stress biomarkers and increasing eNOS expression in the abdominal aorta. Additionally, we report that CFE-treated mice showed reduced weight gain and fat deposition.

Obesity is a complicated medical condition that occurs when a person has an abnormal amount of body fat [41]. Obesity has been linked to a higher risk of developing vascular dysfunction [42], which is a major underlying pathology associated with CVD development. Obesity-related complications, such as type-2 diabetes, atherosclerosis and CVDs, typically take decades to develop, and diet-induced obesity animal models are considered the gold standard for comparison to human obesity-related pathologies [43]. Results from this study show that groups treated with CFE or the combination of CFE + LOR had less weight gain and fat deposition compared to those treated with just a HFD or LOR alone. Fat content in the diet influences body fat accumulation only when energy consumption exceeds energy expenditure [43]. Pregnane glycosides are among the compounds in CFE that are known for their ability to decrease appetite-signaling in the hypothalamus [29,44]. Although the mechanism of action of CFE is unknown, previous studies suggested that it may reduce the production of ghrelin in the stomach and neuropeptide Y in the hypothalamus [28,44]. In comparison to a placebo, CFE maintained body weight, decreased waist circumference, and decreased daily energy intake in overweight adults during a 16-week period. The CFE group’s satiety hormones did not alter because of the change in calorie consumption [28]. The results are in line with previous studies on the influence of CFE on appetite regulation in mice [29] and weight management through reduced waist circumference in human studies [28].

The primary etiology of metabolic syndrome is thought to be central obesity, which is reflected in more recent classifications of the condition, which include a large waist circumference as a prerequisite [45]. Despite the well-documented links between obesity and hypertension, insulin resistance, and CVDs, the underlying mechanisms are not fully understood [46,47]. Adipose tissue (AT) is a dynamic organ found throughout the body, which has an almost limitless ability to increase with obesity [43]. White adipose tissue (WAT) constitutes the majority of whole-body AT and is located in the abdominal cavity and subcutaneously, surrounding important organs and arteries. WAT stores extra energy (in the form of triglycerides), and a higher WAT accumulation, particularly in visceral depots, is a significant promoter of the associated risk for cardiometabolic diseases, hypertension and CVDs [21,48]. However, CVDs may be negatively associated with beige and brown adipose tissue [43]. Vascular dysfunction occurs when arteries lose their capacity to control blood flow adequately, increasing the risk of developing cardiovascular pathologies, such hypertension, atherosclerosis and stroke [43]. Furthermore, childhood obesity has been linked to an increased risk of developing type-2 diabetes, hypertension, dyslipidemia, atherosclerosis and CVDs in adulthood [49]. Long-term high-fat feeding in mice resulted in enhanced adipogenesis and hypertrophy in visceral AT, including mesenteric perivascular AT (PVAT) [49,50], whereas hypertrophy in subcutaneous AT adapted to greater energy intake [49]. PVAT adheres to most blood vessels, notably the aorta and the carotid, coronary and mesenteric arteries [50]. Data from clinical and animal studies show that PVAT is involved in paracrine interaction with blood vessels, as well as physiological homoeostasis and pathological alterations in the cardiovascular system [50]. In ATF3 deficiency, a HFD resulted in increased mean arterial pressure, increased monocyte chemoattractant protein-1 expression and hypertrophy, aberrant fatty tissue formation in the thoracic aortic PVAT and increased vascular wall tension in mice [50]. The obese mice with ATF3 deletion or impairment showed dramatically reduced pancreatic *β*-cell activity, decreased insulin production and increased metabolic dysfunction [51].

The ability of dietary supplementation or obesity to induce CVDs in rodent models without genetic modifications, such as low-density lipoprotein receptor and apolipoprotein E deficiency, has been the subject of significant debate in the scientific literature [52,53,54]. Mice are the most commonly used animal model for cardiovascular physiology and pathophysiology research because they are easy to handle, require minimal maintenance and have low purchase and husbandry costs, despite being phylogenetically distant from humans [52]. There is burgeoning evidence suggesting that administration of a HFD and obesity may be sufficient to invoke endothelial and vascular disturbances in mice; however, HFD composition and duration [54,55], as well as mouse strain [56], age [57] and sex [58] are important to successfully establish diet-induced obesity and subsequent cardiovascular system involvement. In a recent study involving 12-weeks of HFD, impaired vascular reactivity in different arteries from adult male C57BL/6N and /6J mice were reported with significantly reduced ACh-dependent vasodilation responses in abdominal aortae and pudendal arteries but not in penile arteries [59]. These results align with an additional study that demonstrated reduced relaxation in abdominal aortae to ACh in male C57BL/6 mice fed a 16-week HFD from the age of 6 weeks [60]. The results from our study support the claim that a prolonged HFD significantly impairs normal vascular function in the abdominal aorta of mice by reducing ACh-sensitivity, measured by relaxation responses to an ACh dose–response. In contrast, a study investigating the metabolic, vascular and inflammatory responses to a 10-week HFD regimen in male and female C57BL/6 mice found no changes in systolic blood pressure, aortic collagen content, glycated hemoglobin or blood triglycerides in either sex [58]. Conversely, this study failed to show differences in ACh-dependent relaxation in the presence or absence of L-NAME (eNOS inhibitor) or indomethacin (non-selective inhibitor of cyclooxygenase 1 and 2), sodium nitroprusside (endothelium independent) dilation, L-NAME-induced contraction or half maximal contraction of ACh between dietary groups in male or female mice; however, impaired maximal relaxation in male mice fed a HFD was observed [58]. Additionally, a study investigating the effect of various 12-week diets (e.g., standard chow, normal fat, cafeteria and HFD) had on obesity, insulin resistance and vascular dysfunction in male C57BL/6 mice demonstrated diminished ACh-mediated vasodilation in thoracic aortae only when aortic rings were not cleaned and PVAT was left intact [55]. Furthermore, the authors showed enhanced eNOS uncoupling and acetylation, as well as reduced eNOS phosphorylation and NO in the PVAT of obese mice [61,62]. These findings indicate that PVAT may have a dynamic role in driving vascular impairment in diet-induced obese mice and the PVAT dysfunction, rather than obesity itself is responsible for reduced vasodilation responses. Similarly, in this study, we observed a significant reduction in eNOS expression in the endothelium and media of the HFD group. In contrast, eNOS expression was improved with CFE treatment in both the endothelium and media, while CFE and CFE + LOR treatments enhanced eNOS expression, specifically in the endothelium, as shown in Figure 6H,I.

Results from this study report that CFE, rather than LOR, either as a standalone treatment or in combination with CFE, was able to significantly enhance relaxation to ACh in the abdominal aortae from mice fed a 16-week HFD to induce obesity. These results contradict previous findings that related the ability of LOR to restore vasodilation responses to ACh in mice fed a 12-week HFD [63]. While the vascular mechanisms of LOR are elusive, its inability to enhance relaxation and dampen the beneficial effect of CFE may be due to the downstream signaling of its target receptor, 5HT2cR. 5HT2cR, a G-coupled protein receptor that binds serotonin, may have an unknown role in cardiovascular function, as its expression has been demonstrated in endothelial cells of cynomolgus monkeys [64] and in the endothelial and vascular smooth muscle cells of the rat aorta [65]. Furthermore, the activation of 5HT2cR using 1-(3-chlorophenyl)piperazine evokes a hypertensive stress response in non-stressed rats, characterized by elevated mean arterial pressure and heart rate [66]. Importantly, this study also showed that the selective blockade of 5HT2cR by SDZ SER 082 blunted this response [66]. The coexistence of hypertensive state and tachycardia has previously been reported after 5HT2cR activation and appears to indicate sympatho-excitatory drive, the release of vasopressin and the inhibition of baroreflex [67]. Moreover, activation of 5HT2cR using selective agonists SCH23390 and (2A)MK212 caused vasoconstriction responses in isolated rat aortae, which were amplified when rings were first incubated with the angiotensin type 1A and vasopressin V1A receptor agonists, angiotensin II and vasopressin, respectively [65]. Thus, while we did not measure blood pressure in this study, our results suggest that LOR did not enhance ACh-relaxation and was unable to improve HFD-induced vascular dysfunction.

The mice treated with CFE displayed significantly improved ACh-mediated relaxation following a HFD. While the effect that CFE has on vascular function is unknown, the ability of CFE to enhance ACh-dependent relaxation may be in part due to its anti-atherosclerotic properties, which may preserve vascular integrity and function. In male Wistar rats fed a cafeteria diet, treatment with CFE (oral gavage; 25, 50 or 100 mg/kg/day) prevented lipid deposition in the intima of the aortic arch [27]. While this study did not investigate endothelial dysfunction, the ability of CFE to prevent atheroma formation suggests that it may have a beneficial effect during atherosclerotic disease development and progression. Additionally, the ability of CFE to enhance ACh-mediated dilation may be through reducing oxidative injury, a key pathophysiological mechanism that drives the initiation CVDs and cardiac complications during obesity [42]. This study found that male Wistar rats fed a 90-day HFD and treated with CFE (200 mg/kg body weight/day) had lower cardiac total lipids, triglycerides, total cholesterol and free fatty acids, increased activity of cardiac antioxidant enzymes (e.g., glutathione peroxidase, glutathione reductase, glutathione-s-transferase, superoxide dismutase, and catalase) and reduced myocardial necrosis, fat deposition and inflammation, when compared to HFD-fed rats [42]. Overall, the beneficial effects observed with CFE treatment may stem from its multifaceted cardiovascular protective properties, including its ability to reduce oxidative stress, prevent atheroma formation and restore ACh-induced relaxation.

Cellular redox homeostasis is a crucial and highly dynamic biological safeguard that provides constant surveillance to immediately detect changes in reducing and oxidizing reactions. It detects shifts in redox status towards deleterious states, such as oxidative and nitrosative stress, and realigns metabolic activities to restore redox equilibrium within cells [68]. Oxidative stress is an underlying component of various diseases, including atherosclerosis, CVDs, obesity and type-2 diabetes, which occurs due to an imbalance between oxidative and antioxidant systems, leading to the excessive production of damaging reactive oxygen (ROS) and reactive nitrogen species (RNS) [69]. Nitrosative stress is closely related to oxidative stress and occurs during biochemical reactions between NO and superoxide, resulting in the generation of the highly damaging peroxynitrite anion [70,71]. Peroxynitrite then selectively interacts with protein-bound and free tyrosine residues causing post-translation modifications (referred to as protein nitration), which result in the production of NT, endothelial and vascular dysfunction, and initiate a cascade of events that are detrimental to cell survival (e.g., lipid peroxidation, deoxyribonucleic acid strand breakage, damage to the cell membrane and activation of cell death pathways) [40,70,71]. We report the harmful effect that HFD-induced obesity in mice has on the vasculature, as shown by the significantly increased expression of NT in the endothelium and media of abdominal aortae. These results align with previous publications that utilize NT as an indirect marker of measuring oxidative and nitrosative stress in the skeletal muscle [72], nerve fibers [73] and renal tissue [74] of HFD-induced obese mice, as well as in the plasma [75] and placental tissue [76] of obese individuals. In line with the literature, the current data, as shown in Figure 7H, demonstrate that HFD-induced stress leads to increased NT expression in the HFD group. This elevated NT expression is significantly regulated by CFE and CFE + LOR treatments. Notably, CFE alone reduced NT expression in the endothelium by nearly 50%.

Importantly, we report the ability of CFE to markedly reduce NT in the endothelium and decrease NT in the media and adventitia (not statistically significant). To date, the ability of CFE to reduce nitrosative stress or NT has not been investigated in the literature; however, CFE has been shown to reduce oxidative stress and promote antioxidative effects in the liver [77], kidney [78], testes [79] and pancreas [80] of mice fed a HFD to induce insulin resistance. Treatment with CFE (200 mg/kg/day) in male Wistar rats fed a 90-day HFD decreased glutathione levels and ameliorated the increase in lipid oxidation and protein oxidation, commonly observed during HFD regimen, in hepatic, renal, testicular and pancreatic tissue [77,78,79,80]. Additionally, CFE treatment reduced glutamate oxaloacetate and glutamate pyruvate transaminases levels in kidney tissue [78], prevented the decline in activity of catalase, glutathione peroxidase, glutathione reductase, glutathione S-transferase and sodium dismutase in pancreatic and testicular tissue [79,80] and diminished the activity of aldose reductase and sorbitol dehydrogenase in the testes [79] of HFD rats. While the ability of CFE to reduce NT is unknown, the individual biochemical compounds within CFE may explain the potential anti-nitrosative capacity of CFE treatment and its beneficial vascular effect. Phytochemical constituents of CFE, such as flavonoids, flavonol and pregnane glycosides, have been identified as containing antioxidant and cardiometabolic risk lowering effects [81] and are key components contributing to the protective effects of CFE against HFD-induced metabolic alteration [78]. Flavonoids are natural substances found in vegetables, fruits, nuts and seeds that contain a variety of beneficial health effects, including anti-inflammatory, antioxidant, antihypertensive, antiplatelet and anti-ischemic effects [82]. A comparative study investigated the anti-nitrosative, antioxidant and cytotoxic activity of eight flavonoids from different subclasses found that all flavonoids were potent RNS and ROS scavengers and that the formation of NT was significantly reduced in a dose-dependent manner [83]. The flavonoid luteolin has been reported to significantly increase NO levels, improve the bioavailability of endothelial prostacyclin (a potent vasodilator and anti-thrombotic), decrease ROS and reduce the expression of NT residues in rat venous endothelial cells, dose-dependently [84]. Correspondingly, luteolin directly induces a relaxation response in a dose-dependent manner in the thoracic aorta segments from male Sprague–Dawley pre-contracted with either phenylephrine or potassium chloride [85]. Moreover, when thoracic aortae were pre-incubated with an eNOS inhibitor, L-NAME, the dilation responses were attenuated [85]. The study also showed that in both rat thoracic aortae and human aortic endothelial cells, eNOS Ser^1177^ and NO production were increased [85], thus suggesting that the action of luteolin is at least in part mediated through eNOS activity.

The endoplasmic reticulum (ER) is responsible for protein synthesis and transport, as well as for proteostasis (protein homeostasis). HFD-induced obesity can lead to ER stress in various tissues, including the hypothalamus, adipose tissue, and liver [86]. Increasing evidence indicates that ER homeostasis interruption generates a feedback mechanism aimed at preventing the collection of misfolded proteins within the ER lumen [87]. This response, known as the unfolded protein response (UPR), promotes normal cellular function by improving the production of chaperone proteins, such as GRP78. Mitophagy induced by the ER stress chaperone GRP78 contributes to obesity [88]. ER stress in the subfornical organ can elevate the activity of neurons in the PVN, possibly resulting in the increased sympathetic stimulation of the liver. This can lead to notable disturbances in the regulation of hepatic glucose and lipid metabolism within the liver [89]. GRP78 expression is markedly upregulated under ER stress conditions [90] and shows a regulatory role in insulin resistance associated with diet-induced obesity, which is closely linked to obesity, type-2 diabetes, and CVDs [18]. In the HFD group, GRP78 mRNA expression was significantly increased by 5.8-fold. In contrast, treatment with Allium macrostemon extract subsequently reduced this expression by 66.9% [91]. In parallel, the present results (Figure 8H,J) show that CFE and CFE + LOR treatments significantly reduced GRP78 expression in both the endothelium and adventitia. Moreover, in the obese individuals, serum concentrations of GRP78 and mRNA expression levels in both subcutaneous and omental adipose tissues were significantly higher than those observed in healthy controls [92]. Our results demonstrate that a HFD used to induce obesity in mice significantly increases the protein expression of GRP78 in the endothelium, media and adventitia of abdominal aorta. Treatment with CFE either alone or in combination with LOR was able to markedly reduce GRP78 expression in the endothelium and adventitia. Again, while the ability of CFE to restore ER homeostasis and reduce GRP78 has yet to be elucidated, its individual components may explain results from our immunohistological studies. Quercetin, a potent antioxidant flavonoid, has been demonstrated to ameliorate ER stress and reinstate ER homeostasis in human umbilical vein endothelial cells [93] and RAW264.7 macrophages [94] by downregulating genes associated with ER stress, GRP78 and C/EBP-homologous protein (CHOP). Furthermore, a study investigating the ability of the flavonol 3′,4′-dihydroxyflavonol (DiOHF) to attenuate ER stress-induced endothelial dysfunction in male C57BL/6J mice treated with tunicamycin (ER stress inducer) reported the ability of DiOHF to inhibit ER stress [95]. DiOHF-treated mice had significantly reduced systolic and diastolic pressure, diminished weight loss, normalized ACh-dependent relaxation, decreased GRP78, CHOP and NADPH oxidase 2 and increased eNOS phosphorylation [95].

## 5. Conclusions

To our knowledge, this study is the first to directly assess the impact of CFE on vascular function. Our findings suggest that CFE can prevent weight gain, reduce weight and fat deposition, and enhance ACh-mediated relaxation by increasing eNOS levels and reducing NT and GRP78. While we observed some potentiating effect of LOR on CFE-induced weight reduction, there was no additional benefit on vascular function. These results indicate that CFE may be a promising natural product for improving vascular function in obesity-related cardiovascular conditions.

## Figures and Tables

**Table 1:** Composition of jelly cubes.

Description	Animal Groups				
	Control	HFD	HFD + CFE	HFD + LOR	HFD + CFE + LOR
Gelatine (%)	7.5	7.5	7.5	7.5	7.5
Saccharine (mg/mL)	1	1	1	1	1
CFE (mg/kg bwt)	-	-	100	-	100
LOR (mg/kg bwt)	-	-	-	5	5

Abbreviations: bwt, body weight; CFE, *Caralluma fimbriata* extract; HFD, high-fat diet; LOR, lorcaserin.

## Data Availability

The data underlying this study are not publicly available due to commercial and IP value. The data are available from the corresponding author upon reasonable request.

## Supplementary Materials

The following supporting information can be downloaded at: https://www.mdpi.com/article/10.3390/nu16244296/s1, Figure S1: Raw isometric tension analysis trace of abdominal aorta form control mouse.
