# Supplementary material for: Caralluma fimbriata Extract Improves Vascular Dysfunction in Obese Mice Fed a High-Fat Diet
Source: Nutrients. 2024 Dec 12;16(24):4296. doi: 10.3390/nu16244296 (PMC11678847; doi:10.3390/nu16244296)
Supplement: Supplementary file 1 [file nutrients-16-04296-s001.zip › nutrients-3321158-supplementary.pdf]

nutrients

MDPI

Supplementary Material

Caralluma fimbriata extract improves vascular dysfunction in obese mice fed a high fat diet

Venkata Bala Sai Chaitanya Thunuguntla[1†], Laura Kate Gadanec[1†], Catherine McGrath[1], Joanne Louise Griggs[1], Puspha Sinnayah[1], Vasso Apostolopoulos[1,2], Anthony Zulli[1*] and Michael L. Mathai[1*]

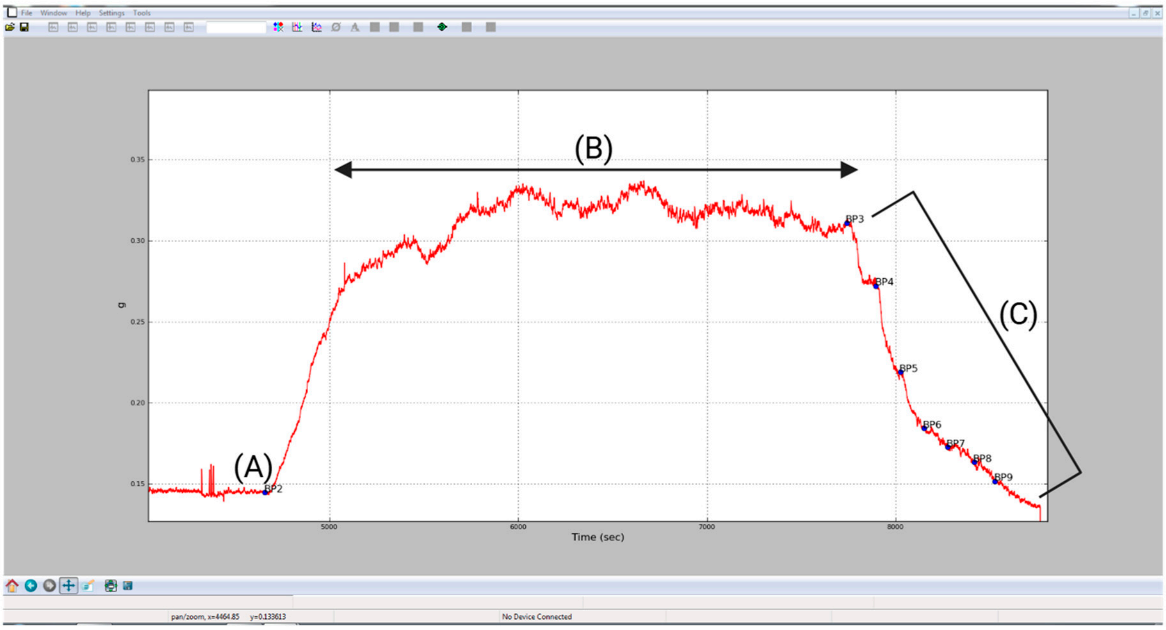

Supplementary Figure S1. Raw isometric tension analysis trace of abdominal aorta from healthy control mouse. Once abdominal aorta rings were acclimatized and stretched to a resting basal tension, **(A)** the thromboxane analog U44619 [2x10^{-5} M] was added to organ baths to cause a contraction response, which resulted in at least a double from baseline tension. **(B)** Rings were then left until a plateau was achieved. **(C)** At this time, an ACh dose-response was then performed [10^{-8} M – 10^{-5}M], with each cumulative dose being added after 2 minutes.
